# Customs, Fashions and Colds

**Published:** 1921-05-28

**Authors:** 


### A NOTE ON OVERCLOTHING.

### Customs, Fashions and Colds.

Apropos of recent exhortations to adopt linen or cotton garments next to the skin, a correspondent to *The Times* has complained that, by contracting violent chills, he has generally paid the penalty for any boldness in this direction. He is by no means alone in his experience, but nevertheless there is much to be said for the use of lighter bodily coverings than we generally affect. In recent times, no one has better explained the situation than Professor Leonard Hill in his comments on the science of ventilation and open-air treatment; but his writings are necessarily somewhat technical, and one hesitates to suggest that the casual reader will, from their perusal, arrive at the truly practical application of scientific facts. Therefore we venture, as a contribution to the discussion, to express in simpler terms some outstanding points in this writer's conclusions.

### Clothing and Bodily Tone.

To begin with, the essential effects of clothing must be understood. In civilised and particularly industrial life it is to be remembered that when man amplifies his powers of heat regulation by means of clothes and artificial heat, he enormously husbands his bodily heat resources and also saves food. By their huddling together in the close atmosphere of a one-room tenement the poor actually do this. In short, they cannot afford a healthy open-air life. But the direct result of such existence is the gradual atrophy from disease of an originally perfect heat-regulating mechanism. The result may be essentially the same in any social sphere. It matters not whether the absence of wages or a chronic parental fear of cold, draughts and wet feet, is the immediate cause. If, first as the child and then as the adult, a person is not in the habit of reasonably maintaining his bodily temperature by natural means, then the inevitable result is weakening of his heat regulating powers, the wearing of heavier and yet heavier clothing and a susceptibility to chills of all kinds. The person who has reached middle age constantly under such conditions can hardly expect to take liberties with the thickness and texture of his clothing.

We must realise the conditions in town life. Instances of the semi-naked savage or the hardy Highlander typify the perfection of the bodily heat regulation, but they cannot justifiably be brought into the discussion. We must admit that, for generations, the average townsman has lost this perfection. Instead, for sedentary occupation he secures a temperature approximating to 33° C. around his skin, and is constantly trying to remove himself from the influence of cold which would increase the need for heat production. In short, his daily life is spent practically under the influence of a tropical climate, and only about 20 per cent. of his body surface is exposed to the air. Admitting that on certain, perhaps many, days of our irregular English climate this heavy protection may be necessary, yet there is no doubt that it would be far preferable to leave a greater margin of protective work for our own bodies to perform. Overwarm clothing provokes an excessive evaporation from the skin, and keeps it in an active state unnecessarily long. The health and bodily tone of all is depressed by overclothing, whereas if that tone is maintained, it is only the underfed who need suffer from scanty clothing. The slight difference between the winter and summer pelts of the warm-blooded domestic animals, such as the horse and cow, clearly indicates the small degree of extra clothing which is required to face an open-air life. In them the intrinsic power of conserving and regulating heat production is intact.

### The Overclothed Child.

And therein lies the whole moral of the discussion. We must not overclothe the child. From the first the natural resources must be developed, otherwise it is impossible without risk, after years with one habitual weight of clothing, to reduce the artificial protection of the adult. Nature's own teaching is all that should be followed. Both wild and domestic animals removed to unaccustomed climates immediately increase or decrease their heavy coats accordingly. "The horse," says Carlyle, "is his own sempster, and weaver, and spinner; nay, his own bootmaker, jeweller, and man-milliner. . . . A perennial rainproof of court-suit on his body wherein warmth and easiness of fit have reached perfection. . . . While I, good heaven, have thatched myself over with the dead fleeces of sheep, the bark of vegetables, the entrails of worms, the hides of oxen or seals, the felt of furred beasts. . . . A moving rag-screen overheaped with rags and tatters raked from the charnel house of Nature." Well may Professor Hill urge that clothes be fashioned to give the greatest adjustability to climate change, and allow man to work not only with ease but with the greatest bodily efficiency. When this is intelligently and generally done there will be no need to deplore the fact that we so easily catch cold.

Clothing as a whole must be considered. Let us realise that it is certainly unnecessary always to wear this or that particular fabric. We should simply choose correctly as the occasion demands. Also, when we revert to the popular questions, such as "wool versus cotton," etc., we should not be discussing them as habits to be acquired in middle age, but as those possibly to be encouraged in earliest youth. Most general writers, in urging the claims of different systems of clothing, have based their deductions on the nature of the material—on the type of fibre. In actual fact, the properties of a garment depend almost wholly, not on the fibre, but on the manner of its weaving. The same results can be secured with wool, cotton, or linen by weaving out of each a material with the same porosity, air-holding power, permeability to moving air and water-evaporating power. Habitually we find wool next to the skin warmer than the customary thin linen garment. But weave the linen equally thick and porous (air-holding) as the wool, and it is just as warm. Thickness for thickness, dry cotton flannelette is no less warm than flannel. One specifies the dryness because wet fabrics have a different action, but the townsman's clothes are rarely wet in the accepted sense. In his case, therefore (though it is obviously not true for extreme climates), he has one problem only to consider—namely, how not to overclothe himself; how to resist a temptation inseparable from a sedentary life. When this has been achieved he need no longer live in dread of the chill-producing propensities of the ordinary cotton vest.

